# Development of a Controlled Release of Salicylic Acid Loaded Stearic Acid-Oleic Acid Nanoparticles in Cream for Topical Delivery

**DOI:** 10.1155/2014/205703

**Published:** 2014-01-21

**Authors:** J. O. Woo, M. Misran, P. F. Lee, L. P. Tan

**Affiliations:** ^1^Department of Chemistry, Faculty of Science, University of Malaya, 50603 Kuala Lumpur, Malaysia; ^2^Mechatronics and BioMedical Engineering, Faculty of Engineering & Science, Universiti Tunku Abdul Rahman, Setapak, 53300 Kuala Lumpur, Malaysia; ^3^Prime Oleochemical Industries Sdn. Bhd., Taman Perindustrian Jaya, 47301 Petaling Jaya, Selangor, Malaysia

## Abstract

Lipid nanoparticles are colloidal carrier systems that have extensively been investigated for controlled drug delivery, cosmetic and pharmaceutical applications. In this work, a cost effective stearic acid-oleic acid nanoparticles (SONs) with high loading of salicylic acid, was prepared by melt emulsification method combined with ultrasonication technique. The physicochemical properties, thermal analysis and encapsulation efficiency of SONs were studied. TEM micrographs revealed that incorporation of oleic acid induces the formation of elongated spherical particles. This observation is in agreement with particle size analysis which also showed that the mean particle size of SONs varied with the amount of OA in the mixture but with no effect on their zeta potential values. Differential scanning calorimetry analysis showed that the SONs prepared in this method have lower crystallinity as compared to pure stearic acid. Different amount of oleic acid incorporated gave different degree of perturbation to the crystalline matrix of SONs and hence resulted in lower degrees of crystallinity, thereby improving their encapsulation efficiencies. The optimized SON was further incorporated in cream and its *in vitro* release study showed a gradual release for 24 hours, denoting the incorporation of salicylic acid in solid matrix of SON and prolonging the *in vitro* release.

## 1. Introduction 

Solid lipid nanoparticles (SLNs) are colloidal particles composed of lipids. They are colloidal carrier systems that are usually applied in the area of controlled drug delivery and cosmetic and pharmaceutical applications [1–3]. The rapid development in SLN research is due to their negligible toxicity and biocompatibility with the human body. Besides, the preparation method is more environmentally friendly as less organic solvent is required [4].

Although SLN demonstrated several advantages as excipient, it does have limitation especially low encapsulation capacity and expulsion of drug during storage which led to the development of new generation of lipid nanoparticles which is also generally known as nanostructured lipid carriers (NLCs) [4]. NLC are SLN incorporated with liquid oil to improve encapsulation efficiency and reduce drug expulsion. Incorporation of liquid oil into solid lipid matrix could lead to a massive crystal lattice disturbance and leaves enough space to accommodate drug molecules, and thus, improve active ingredient encapsulation efficiency [2, 5].

SLN and NLC can be prepared from different types of starting material such as triglycerides, phospholipids, and waxes [6–9]. Among them, fatty acid is one of the most cost effective raw materials and therefore has been widely used in order to reduce the production cost. Therefore, fatty acids were used as raw material in the preparation of SONs in order to develop a cost effective carrier system.

Salicylic acid is a common active ingredient used in topical formulation [10] for therapeutic treatment such as acne due to its keratolytic property [11]. However, it may cause a mild to strong skin irritation to certain patients [12]. The reduction of antiacne agent irritancy through incorporation in the sustained release formulations such as liposome, microemulsion, hydrogel, and SLN has been reported [13]. Hence, encapsulation of salicylic acid in the prolonged release delivery system could be a potential approach to minimize its side effects and reduce the application frequency, thus offering better patient compliance.

In the present work, a series of salicylic acid loaded SONs incorporated with different ratio of oleic acid (OA) were prepared to investigate the optimum encapsulation efficiency. The impact of OA and salicylic acid incorporated in SONs on their physicochemical properties, such as morphology, particle size, and thermal analysis, were studied. Then, an optimized SON formulation was further incorporated in the base cream to assess the *in vitro *release of salicylic acid.

## 2. Materials and Methods

### 2.1. Materials

Stearic acid and phosphate buffer saline (PBS) tablets (pH 7.4) were purchased from Sigma-Aldrich (St. Louis, USA). Oleic acid was obtained from Fluka (Buchs, Switzerland). Tween 60 (polyoxyethylene sorbitan monostearate) was obtained from Lasem Asia (Kuala Lumpur, Malaysia). Salicylic acid and methanol were obtained from Merck (Darmstadt, Germany). Salcare SC91 was obtained from BASF (Ludwigshafen, Germany). All solutions and samples were prepared by using deionized water with a resistivity of 18.2 *Ω* cm^−1^, supplied from a Barnstead Diamond Nanopure Water Purification unit coupled with a Barnstead Diamond RO unit (Barnstead International, USA).

### 2.2. Preparation of SONs

SONs were prepared by melt emulsification method combined with ultrasonication technique. The lipid phases, stearic acid (0 wt% OA), or mixture of OA and stearic acid with 10, 20, 30, 40, and 50 wt% OA ratio, respectively, were prepared and melted at 75°C. The molten lipid phases were mixed with preheated Tween 60 solutions. The mixtures were homogenized by homogenizer (SilentCrusher M, Heidolph Instruments, Germany) and then further treated by ultrasonic liquid processor model XL 2015 (USA) to form nanoemulsions. The nanoemulsions were poured into 20 mL of cold water (about 2°C) to form SON dispersions. Salicylic acid loaded SON dispersions were prepared exactly in the same manner only adding 10 mg salicylic acid into lipid phases above. All SON dispersions were stored at 5°C after preparation.

### 2.3. Transmission Electron Microscopy (TEM)

The morphological observations of SONs were obtained by using Energy Filtered TEM model LIBRA 120 equipped with an Olympus SIS-iTEM (version 5). A drop of SON dispersion was placed onto a carbon-coated copper grid followed by the removal of excess dispersion using a filter paper. It was then negatively stained using 2% phosphotungstic acid solution and air-dried at room temperature for 30 min. The grid was then ready to be examined under TEM.

### 2.4. Mean Particle Size and Zeta Potential of SONs Measurement

The average particle size and zeta potential of SONs were determined by using ZetaSizer ZS (Malvern Instruments, UK). SON dispersion was diluted with deionized water and was equilibrated to room temperature for 10 min. A 1 cm path length clear quartz cuvette was used for the particle size measurement. Zeta potential measurement was carried out using disposable folded capillary cell. Both measurements were performed at a constant temperature of 25°C.

### 2.5. Differential Scanning Calorimetry (DSC) Analysis

Thermal behaviours of SONs were characterized by using Pyris 6 DSC (Perkin-Elmer, USA). SON dispersions were dried in a desiccator for 24 hours prior to DSC analysis. About 6 mg of air-dried SON sample was weighed into 40 *μ*L aluminium sample pan. An empty sample pan was used as a reference. The heating run was performed from 35°C to 80°C with the heating rate of 5°C min^−1^ by continuously flushing the nitrogen gas at the rate of 20 mL min^−1^.

### 2.6. Encapsulation Efficiency of Salicylic Acid Loaded SONs

Encapsulation efficiency of salicylic acid loaded SON was determined using ultrafiltration method. SON dispersion was placed into the upper chamber of centrifugal filter tubes with 50000 Da molecular weight cut-off (Vivaspin 6, Sartorius Stedim Biotech, Germany) and centrifuged for 30 min at 8000 rpm. Then, the amount of free salicylic acid in the filtered aqueous phase was diluted and determined spectrophotometrically (Cary 50 UV-Vis Spectrometer, Agilent Technologies, USA) at wavelength of 297 nm and the concentration of salicylic acid in each sample was determined from a standard concentration curve (1 µg mL^−1^–25 µg mL^−1^). Encapsulation efficiency was calculated by the following equation:

(1) $$\begin{array}{c} \text{EE}=\left(\frac{W_{T}-W_{F}}{W_{T}}\right)\times 100\%, \end{array}$$

where EE is encapsulation efficiency of SON, *W*_*T*_ is the weight of salicylic acid added during preparation, and *W*_*F*_ is the weight of unloaded salicylic acid filtered aqueous phase, respectively.

### 2.7. Sample Preparation

Four different samples were prepared in order to evaluate the *in vitro* release. The four different samples, salicylic acid solution, salicylic acid cream, salicylic acid loaded SON and salicylic acid loaded SON, incorporated creams were denoted as Samples A, B, C, and D. Sample A was prepared by dissolve salicylic acid in deionised water. Sample B was prepared by homogenizing the salicylic acid solution and Salcare SC91 base cream obtained from BASF, a raw material that consisted of 8% anionic acrylic copolymer dispersed in a 5–8% of medical grade white oil. Sample C is the optimized SON dispersion and this SON dispersion was further formulated into Salcare SC91 base cream at the homogenization rate of 5000 rpm for 5 min to prepare Sample D.

### 2.8. *In Vitro* Release

The *in vitro* drug release of the four different samples were evaluated by using Automated Franz Diffusion Cell System (Microette Autosampling System, Hanson Research Co., USA) with 0.636 cm^2^of effective diffusion area and 4 mL of diffusate chamber volume. The diffusate compartments were filled with a 10 mM PBS solution (pH 7.4), stirred at 400 rpm, and thermostated at 37 ± 1°C during all the experiments. Regenerated cellulose membranes with a 5000 Da molecular weight cut-off were used in these experiments. Pretreatment of the membranes by soaking in the receiving medium were performed for 1 hour before mounted to the Franz Diffusion Cells. Approximately 1 g of each sample was placed on the membrane surface in the retentate compartment. Each experiment was run using six different retentate compartments for 24 hours. At predetermined intervals, samples were withdrawn and replaced with fresh receiving medium. The samples were spectrophotometrically analyzed for the drug content as described earlier. Each data point was as the average of six measurements.

## 3. Results and Discussions

### 3.1. Morphology

**Figure 1:**
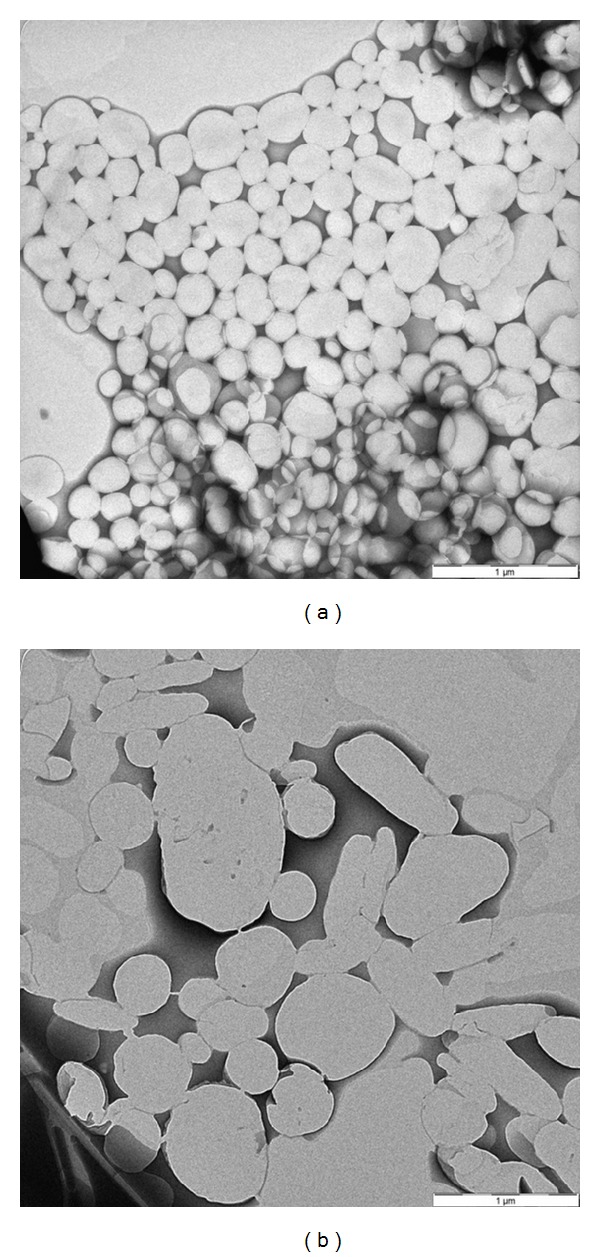
The TEM micrographs of SONs. (a) SON without OA and (b) SON with 30 wt% OA composition.

The morphology of SON without OA appeared in monodisperse spherical shape with small particle size as shown in Figure 1(a). On the contrary, the TEM micrograph revealed that the higher OA composition (30 wt%) led to the formation of elongated particles with larger particle size (Figure 1(b)). This morphological observation was in agreement with the result obtained from particle size measurement of SONs. The presence of elongated particles is also directly related to the increase in polydispersity indexes of lipid nanoparticles.

### 3.2. Particle Size of SONs

The particle size of SONs prepared by this method was investigated under Dynamic Light Scattering technique. It was observed that the mean particle size of SON depended on the amount of OA in the mixture. The average particle size of unloaded SONs increased from 194 nm to 255 nm with increasing OA amount up to 30 wt%, while it decreased by about 14% when the OA composition achieved up to 50 wt% (Table 1). SON with salicylic acid also showed the similar trend for the results of mean particles size. These observations revealed that the excipients' mean particle sizes varied with the amount of OA in the mixture. However, the decrease in size of the particles with higher amount of OA (>40 wt%) may be due to the incompatible mixing between OA and stearic acid. The free OA might form nanoemulsion with excess surfactant which results in formation of smaller particles and consequently gives higher polydispersity index.

The mean particle sizes of the salicylic acid loaded SON in general increased from 20 to 40% as compared to its unloaded SON (Table 1). Incorporation of salicylic acid in SONs also resulted in higher polydispersity indexes. The significant increase in mean particle size and polydispersity index when salicylic acid was added in the SON indicated the salicylic acid participation in the formation of lipid nanoparticles.

### 3.3. Zeta Potential of SONs

The zeta potential for all SON dispersions was found to be lower than −40 mV (Table 1). Generally, suspended particles are considered to be stable when absolute value of zeta potential is above 30 mV [14]. Although the measurement of zeta potential allows predictions about the storage stability of colloidal dispersion, this rule cannot strictly applied for systems which contained steric stabilizer [15]. Since Tween 60 contained poly(ethylene oxide) moiety on its polar head group, its electrostatic repulsion effect was negligible as compared to its steric repulsion effect. Unlike ionic surfactant, Tween 60 could not ionize in aqueous solution, but it induces hydroxyl ion adsorption on the polar nonionic head group of the surfactant molecule. This water molecules layer was absorbed onto the surface of the particle and formed a repulsion barrier layer to stabilize the particles of SON [9].

### 3.4. Differential Scanning Calorimetry

**Figure 2:**
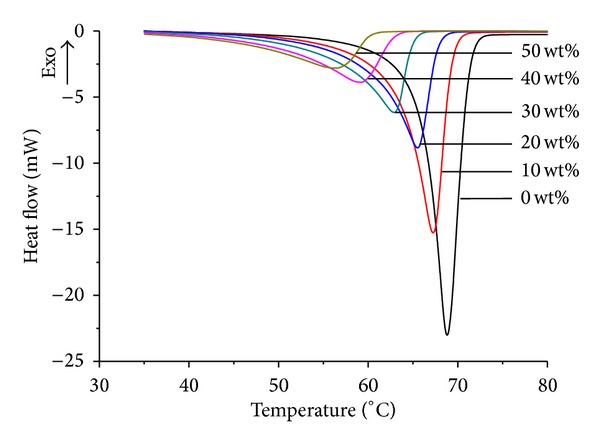
DSC thermograms of salicylic acid loaded SONs with 0–50 wt% OA ratio.

All the DSC curves of salicylic acid loaded SONs showed one endothermic peak and the melting point shifts towards lower temperature accompanying the increase in width of the melting peak (Figure 2). The melting point of SON gradually decreased by about 12°C from 0 wt% to 50 wt% OA ratio (Table 2). This is due to the massive crystal matrix disturbance in SONs. The degree of crystallinity of SONs was determined by calculating the ratio of SON enthalpy to stearic acid enthalpy as shown by the following equation:

(2) $$\begin{array}{c} \text{CD}=\left(\frac{H_{\text{SON}}}{H_{\text{SA}}}\right)\times 100\%, \end{array}$$

where CD is degree of crystallinity of SON, *H*_SON_ is enthalpy of SON, and *H*_SA_ is the enthalpy of pure stearic acid, respectively. Taking the enthalpy of stearic acid at 226 J g^−1^ as 100%, the theoretical percentage of crystallinity of SONs with 10, 20, 30, 40, and 50 wt% OA could then be obtained [16]. The degree of crystallinity of SONs was dramatically reduced about 67% with increasing OA ratio from 0 wt% to 50 wt% (Table 2). These could conclude that the presence of OA perturbs the stearic acid crystal matrix and increases amorphous proportion in particles, leading to reduction in the degree of crystallinity of SONs.

### 3.5. Encapsulation Efficiency of SON

**Figure 3:**
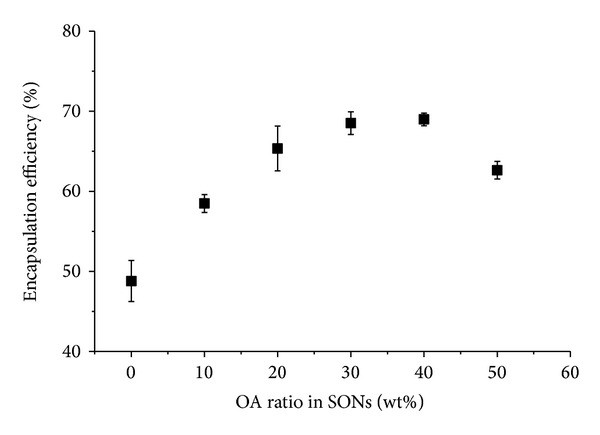
Encapsulation efficiency of salicylic acid loaded SONs with different OA composition.

The encapsulation efficiency of SONs increased from 49 to 69% with increasing the OA composition from 0 to 40 wt%, respectively, but slightly decreased to 63% for 50 wt% OA ratio as shown in Figure 3. Encapsulation efficiency was always correlated with the crystallinity degree of lipid nanoparticles [17]. It is important to note that the more OA in the mixture the higher the encapsulation efficiency. This is because incorporation of OA increases the amorphous proportion in the solid lipid matrix and as a result decreases the overall particle crystallinity, thereby improving the encapsulation efficiency. Jenning et al. [7] also reported that incorporation of liquid oil into lipid nanoparticles perturbed the crystalline matrix resulting in enough space to accommodate retinol molecules and thus increased the drug encapsulation capacity. This is further supported by Morselli Ribeiro et al. [18] who reported that encapsulation efficiency of lipid nanoparticles was directly proportional to the oleic acid concentration in the lipid phase. However, the encapsulation efficiency of SONs containing large amount of OA (50 wt%) slightly decreased. This is due to the exclusion of OA during crystallization of SONs and subsequently affect the incorporation of salicylic acid in SONs [19, 20]. Both SONs with 30 wt% and 40 wt% OA composition demonstrated high loading efficiency of salicylic acid with similar sizes but SON with 30 wt% OA ratio was considered as an optimized formulation because SON with higher oleic acid amount has lower melting behaviour and may be easily oxidized especially when there is unexpected temperature fluctuation during storage and transportation.

### 3.6. *In Vitro* Release

**Figure 4:**
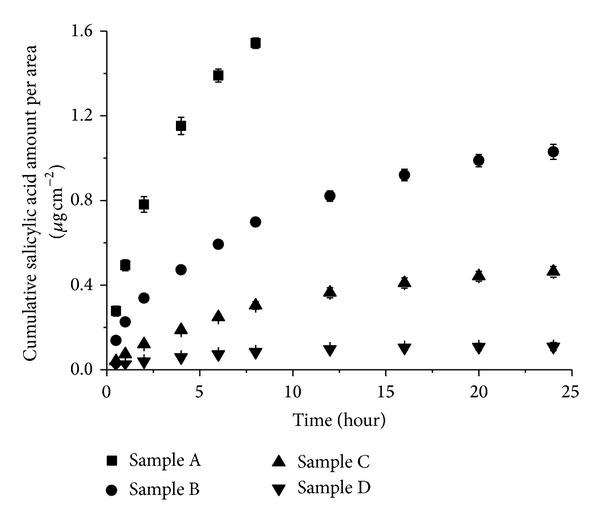
*In vitro* release of four different samples over a period of 24 hours.

SON with 30 wt% OA ratio was selected as the optimized formulation based on the results obtained from our particle size, thermal behavior, and encapsulation efficiency analysis. It was then further incorporated into a cream formulation for the *in vitro* release investigation. Four different samples as described in Section 2.7 were evaluated using Static Franz Diffusion Cell method and the cumulative salicylic acid release from all samples was plotted against time (Figure 4). Sample A showed a rapid released within 8 hours whereas Samples B, C, and D demonstrated a biphasic release pattern whereby a rapid release of salicylic acid was observed in the first 8 hours followed by slower release at almost a constant rate. The initial fast release rates of Samples B, C, and D could be due to the presence of free salicylic acid molecules in the aqueous phase which diffuses rapidly through the membrane. After the completion of the first phase of free salicylic acid within the first 8 hours, it is followed by the second phase which has much slower release rate that could be due to the retention of salicylic acid in the SON particle dispersion and/or in the emulsion. Samples C and D have slower release behaviour as compared to the samples without SON. The presence of controlled release behaviour for samples containing SON (Sample C and D) revealed that the salicylic acid molecules were successfully incorporated in the solid matrix of SON and slowly released from the particles.

**Figure 5:**
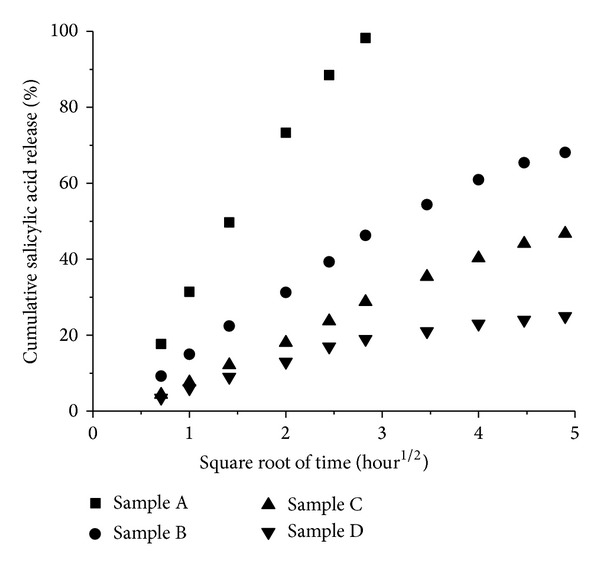
Cumulative release of ■ salicylic acid solution, ● salicylic acid cream, ▲ salicylic acid loaded SON with 30 wt% OA ratio, *▼* salicylic acid loaded SON with 30 wt% OA ratio in cream plotted against square root of time at 37°C, respectively.

**Figure 6:**
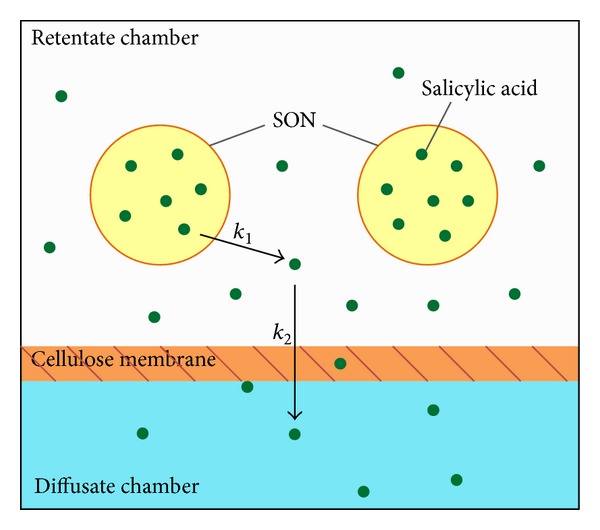
Schematic diagram for *in vitro* release mechanism of salicylic acid loaded SON in Franz Diffusion Cell.

The *in vitro* releases of the four different samples were curve-fitted to zero order, first order, and Higuchi model in order to understand their release kinetics. We have chosen the Higuchi model because it could describe the diffusion of drug from homogenous and granular matrix system [21]. The release profiles of the four samples showed the best fit into Higuchi model (*R* ^2^ > 0.95) (Table 3). Linear fits for all samples were obtained (Figure 5), denoting that the release of salicylic acid from the samples was diffusion controlled process [22]. The slopes obtained from the plotting of Higuchi model (Figure 5 and Table 3) represent the release rate of salicylic acid [23]. The release rate among the four samples was A > B > C > D. The release rate of Sample A was reduced when formulated into cream (Sample B) revealing this retentional effect by the emulsion. The release rate of Sample B is further decreased to about 3 times when salicylic acid loaded SON was incorporated into cream (Sample D) and this is indicating that the SON prepared in this study has a slow release property. This slow release property of SON could be due to the release of salicylic acid from the solid matrix of SON particle which involved two pathways as illustrated in Figure 6. In the retentate chamber, the salicylic acid slowly released (*k*_1_) from the particles to the aqueous phase and then diffused through the membrane at the faster rate (*k*_2_) (Figure 6). The reason of slow release of salicylic acid at the first pathway may be due to the hydrophobic solid matrix of SON retaining the release of salicylic acid to the aqueous phase and thus results an prolonged release rate.

## 4. Conclusion

In this study, melt emulsification method combined with ultrasonication technique was employed to prepare a series of cost effective salicylic acid loaded SONs dispersion incorporated with different OA ratio. TEM micrographs of SONs showed that SON without OA appeared in small and regular spherical shape whereas incorporation of OA tends to elongate the particles of SONs. Particle size and polydispersity index of SONs were seen to depend on the amount of OA and salicylic acid incorporated. The results of DSC revealed that the presence of OA in stearic acid lipid matrix has perturbed the organization in the crystal lattice, leading to reduction in the degree of crystallinity of SONs and thus improving the encapsulation efficiency. SON with 30 wt% OA ratio was selected as the optimized formulation and further incorporated in cream to investigate its *in vitro* release. The salicylic acid loaded SON prepared in this study has a prolong release property which may be due to the incorporation of salicylic acid in solid matrix of SON. The incorporation of SON into cream formulation further reduced the release rate over a period of 24 hours revealing that the emulsion also slowed down the release of salicylic acid. Our finding suggests that salicylic acid loaded SON enriched cream could be a promising delivery system for the enhancement of the therapeutic efficacy in the topical treatment application.

## Figures and Tables

**Table 1:** The mean particle size, polydispersity index, and zeta potential of freshly prepared unloaded and salicylic acid loaded SONs at 25°C.

OA ratio (wt%)	Unloaded SONs			Salicylic acid loaded SONs		
	Mean particle size (nm)	Polydispersity index	Zeta potential (mV)	Mean particle size (nm)	Polydispersity index	Zeta potential (mV)
0	194 ± 2	0.19 ± 0.01	−45.9 ± 0.9	271 ± 3	0.25 ± 0.01	−39.4 ± 0.5
10	221 ± 4	0.21 ± 0.02	−47 ± 2	283 ± 8	0.29 ± 0.03	−43 ± 1
20	246 ± 3	0.31 ± 0.03	−45.5 ± 0.5	311 ± 7	0.35 ± 0.02	−45 ± 1
30	255 ± 3	0.37 ± 0.02	−50 ± 1	332 ± 3	0.37 ± 0.03	−43 ± 1
40	229 ± 4	0.37 ± 0.01	−43 ± 1	330 ± 10	0.40 ± 0.02	−44.0 ± 0.8
50	223 ± 4	0.36 ± 0.02	−46 ± 1	280 ± 5	0.38 ± 0.02	−45.2 ± 0.7

**Table 2:** Onset temperatures (*T*_onset_), melting points, melting enthalpies (Δ*H*), and crystallinity degree of SONs with and without salicylic acid.

OA ratio (wt%)	Salicylic acid loaded SONs			
	*T*_ onset_ (°C)	Melting point (°C)	Δ*H* (J/g)	Crystallinity degree (%)
0	65.9	68.8	188	83
10	63.1	67.2	158	70
20	59.9	65.5	119	53
30	55.8	62.9	98	43
40	49.0	59.2	78	35
50	44.2	56.5	60	27

**Table 3:** Different kinetic model evaluation of salicylic acid release for four different samples.

Sample	Zero order		First order		Higuchi model	
	Slope	*R* ^2^	Slope	*R* ^2^	Slope	*R* ^2^
A	10.5	0.93	0.20	0.77	38.4	0.99
B	2.4	0.89	0.07	0.69	14.4	0.98
C	1.8	0.92	0.08	0.70	10.5	0.99
D	0.9	0.81	0.02	0.62	5.2	0.95
